# Needs-based triggers for timely referral to palliative care for older adults severely affected by noncancer conditions: a systematic review and narrative synthesis

**DOI:** 10.1186/s12904-023-01131-6

**Published:** 2023-03-09

**Authors:** Arisa Kawashima, Catherine J. Evans

**Affiliations:** 1 Department of Nursing for Advanced Practice, Division of Integrated Health Sciences, Nagoya University Graduate School of Medicine, Nagoya, Japan; 2 King’s College London, Cicely Saunders Institute of Palliative Care, Policy and Rehabilitation, Faculty of Nursing, Midwifery and Palliative Care, London, UK; 3 Sussex Community NHS Foundation Trust, Brighton, UK

**Keywords:** Palliative care, Aged, Systematic review, Referral and consultation

## Abstract

**Background:**

Older people with noncancer conditions are less likely to be referred to palliative care services due to the inherent uncertain disease trajectory and a lack of standardised referral criteria. For older adults with noncancer conditions where prognostic estimation is unpredictable, needs-based criteria are likely more suitable. Eligibility criteria for participation in clinical trials on palliative care could inform a needs-based criteria. This review aimed to identify and synthesise eligibility criteria for trials in palliative care to construct a needs-based set of triggers for timely referral to palliative care for older adults severely affected by noncancer conditions.

**Methods:**

A systematic narrative review of published trials of palliative care service level interventions for older adults with noncancer conditions. Electronic databases Medline, Embase, CINAHL, PsycINFO, CENTRAL, and ClinicalTrials.gov. were searched from inception to June 2022. We included all types of randomised controlled trials. We selected trials that reported eligibility criteria for palliative care involvement for older adults with noncancer conditions, where > 50% of the population was aged ≥ 65 years. The methodological quality of the included studies was assessed using a revised Cochrane risk-of-bias tool for randomized trials. Descriptive analysis and narrative synthesis provided descriptions of the patterns and appraised the applicability of included trial eligibility criteria to identify patients likely to benefit from receiving palliative care.

**Results:**

27 randomised controlled trials met eligibility out of 9,584 papers. We identified six major domains of trial eligibility criteria in three categories, needs-based, time-based and medical history-based criteria. Needs-based criteria were composed of symptoms, functional status, and quality of life criteria. The major trial eligibility criteria were diagnostic criteria (*n* = 26, 96%), followed by medical history-based criteria (*n* = 15, 56%), and physical and psychological symptom criteria (*n* = 14, 52%).

**Conclusion:**

For older adults severely affected by noncancer conditions, decisions about providing palliative care should be based on the present needs related to symptoms, functional status, and quality of life. Further research is needed to examine how the needs-based triggers can be operationalized as referral criteria in clinical settings and develop international consensus on referral criteria for older adults with noncancer conditions.

**Supplementary Information:**

The online version contains supplementary material available at 10.1186/s12904-023-01131-6.

## Background

Inequities in the provision of palliative care remain globally, whilst palliative care should be available to all who need it regardless of their diagnosis [1]. Global ageing and the changes in the prevalence of diseases imply that most needing palliative care worldwide are older people living with noncancer conditions [1–4]. However, there is consistent evidence that older people with noncancer conditions experience inequitable access to palliative care with low rates of referral or late referral in the last days or weeks of life [5, 6], such as in dementia [7] and heart failure [8].

There are several major barriers to referral in patients with noncancer conditions. A systematic review reported that one of the barriers to access and referral to palliative care is ‘a lack of national standardised referral criteria’ for screening patients with chronic noncancer disease regarding their need for palliative care [9]. A questionnaire survey showed that the highest barrier perceived by specialist palliative care service providers was ‘the unpredictable noncancer disease trajectory’ [10]. In primary care settings, the uncertainty of the illness trajectory was also identified as a barrier to effective primary palliative care provision for noncancer patients [11]. As a result, in clinical practice, key triggers or prompts for older adults with noncancer conditions to access palliative care are based on variable professional opinions or experiences [12, 13]. This means referral triggers are typically informed by differences in education, interest, and understanding on the intended outcomes of palliative care.

Referral criteria are needed to address the inequity of access to palliative care for older adults with noncancer conditions. Systematic review on referral criteria for noncancer patients aged over 65 years identified predictor variables to aid clinicians’ prognostic estimation [5]. However, the inherent uncertain disease trajectory for older adults with noncancer conditions requires the provision of palliative care to be based upon need, rather than time-based criteria, such as disease trajectory and prognostic criteria [13, 14]. Although systematic reviews identified referral criteria for palliative care among patients with heart failure [15, 16], dementia [17], and Parkinson’s disease [18], there has been no referral criteria based on palliative care needs for older general noncancer populations and the reviews assert the lack of consensus on palliative care referral criteria for adults with noncancer conditions.

Eligibility criteria for participation in clinical trials on palliative care can inform a needs-based criteria for palliative care. This approach was used and advocated by Hui et al. [12] and others [15, 17, 18] in systematic reviews investigating eligibility criteria for trials to inform triggers for outpatient palliative cancer care. Hui et al.’s review identified six themes for referral, two time-based, including cancer trajectory and prognosis, and four needs-based, including physical symptoms, performance status, psychosocial distress, and end-of-life care planning [12]. As appropriate trial eligibility criteria are designed to measure efficacy of an intervention, the criteria include populations considered likely to benefit from the intervention compared with the control. Eligibility criteria in palliative care trials seeks to identify patients with palliative care needs and considered likely to benefit from palliative care. Although eligibility criteria for randomised controlled trials (RCTs) may not be specifically designed for referral, they can inform a needs-based set of triggers for timely referral to palliative care.

## Aim

This systematic review aimed to identify, appraise the applicability, and synthesise patient eligibility criteria in published trials on palliative care service level interventions for older adults severely affected by noncancer conditions. The findings intended to construct a needs-based set of triggers for timely referral to palliative care.

## Methods

### Study design

A systematic narrative review of the published literature on palliative care interventions for older adults with noncancer conditions to identify and synthesise the criteria used to indicate eligibility for palliative care provision [19]. The review was conducted in accordance with the Preferred Reporting Items for Systematic Reviews and Meta-analyses (PRISMA) 2020 guidelines [20]. The PRISMA 2020 checklist is shown in Additional file 1. The methodological quality of the included studies was assessed using a revised Cochrane risk of bias tool for randomized trials [21]. The protocol was registered in PROSPERO in November 2018 (CRD42018095845, https://www.crd.york.ac.uk/prospero/display_record.php?ID=CRD42018095845845). We were not required to seek an institutional ethics approval because we only used publicly accessible documents.

### Data sources and searches

Relevant articles were identified from six electronic searches: MEDLINE (Ovid), EMBASE (Ovid), CINAHL (EBSCOhost), PsycINFO (Ovid), the Cochrane Central Register of Controlled Trials (CENTRAL), and ClinicalTrials.gov. Search strategies were informed by previous systematic reviews related to palliative care and older adults [12, 13, 22]. A full search strategy can be seen in Additional file 2. All searches were conducted from database inception to September 2018 and updated on June 2022. We supplemented the electronic searches with reference chaining and citation tracking, and handsearching two palliative medicine textbooks [23, 24] and conference abstracts [Research Congress of the European Association for Palliative Care (EAPC), 2018]. All identified studies were managed in EndNote. There was no language restriction in the selection of studies.

### Eligibility criteria

#### Types of studies

We included RCTs, including cluster randomised trials, pilot, and feasibility trials. We sought to identify and collate trial eligibility criteria for patient participants and appraise what patterns of eligibility criteria were successful in terms of recruitment, attrition, attaining sample size, and effect on the primary outcome. Feasibility and pilot trials were included, as intention is to evaluate if they can recruit patients to the trial using the stated eligibility criteria. We excluded trials that focused exclusively on the economic evaluation of palliative care as not evaluating the effect of palliative care on patient outcomes, and non-experimental studies (observational studies) as our interest was patient eligibility criteria for palliative care intervention trials. We excluded opinion pieces including editorials, commentaries, letters, and dissertations.

#### Types of participants

We included adults (aged ≥ 65 years) severely affected by chronic noncancer illness, including chronic heart failure (CHF), chronic obstructive pulmonary disease (COPD), chronic kidney failure, cirrhosis of the liver, stroke and long-term neurological conditions, including dementia and Parkinson’s disease. These conditions can cause considerable distressing symptoms and concerns [25]. Receipt of palliative care is shown to relive suffering [26, 27]. We included studies where > 50% of the population was aged ≥ 65 years and > 50% of the population were people with noncancer conditions.

#### Types of interventions

We included palliative care trials to identify individuals who could benefit from palliative care. Specialist and general palliative care interventions were included that aimed to promote quality of life for adults aged older adults ≥ 65 years severely affected by noncancer conditions. We defined a palliative care intervention as a model or service of palliative care, not a discrete aspect of palliative care, such as oxygen therapy. We defined a palliative care model or service as comprising four key elements [28–31], namely:

##### All levels of palliative care in any setting

We referred to the model of a three-level structure: palliative care approach in all settings, general basic palliative care, and specialist palliative care with adequate skills for each level [32]. All study settings were included: community health services, including clinics and health centres, outpatient and ambulatory care settings, and inpatient units.

##### An intervention providing direct palliative care to older adults

Interventions that did not directly deliver care to patients were excluded (e.g., interventions to caregivers, education programs to healthcare professionals, or evaluations of assessment tools). We considered a palliative care service intervention if the authors described it as 'palliative' anywhere in the manuscript.

##### An intervention had multi-component services

A palliative care service is a multidimensional and holistic approach to meet the physical, psychological, social, and spiritual needs of patients. Interventions that delivered only one component of palliative care (e.g., medication, psychotherapy, complementary therapy, decision aid) were not considered as palliative care service.

##### An intervention was provided by a multidisciplinary team

We defined 'palliative care services' as multidisciplinary services providing comprehensive care aiming at different physical and psychosocial components of palliative care. We excluded interventions provided by only one professional (e.g., nursing intervention).

### Outcome

Types of outcome measures were not restricted.

### Study selection and data extraction

**Fig. 1:**
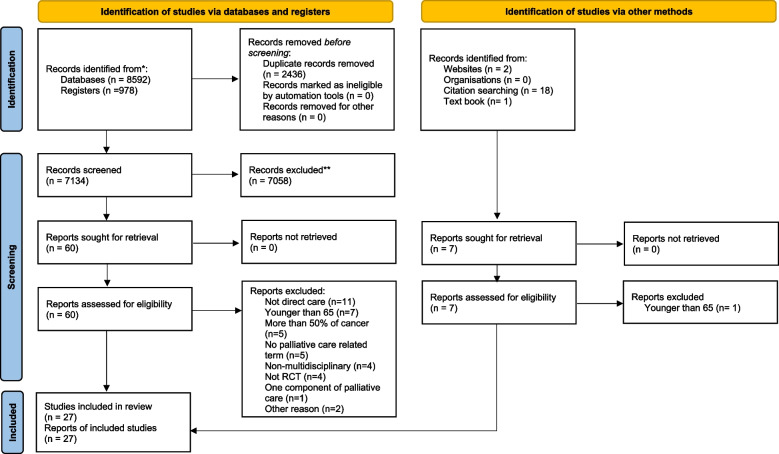
PRISMA 2020 flow diagram

The review author A.K. screened and assessed the identified titles and abstracts according to the inclusion criteria, followed by assessing all relevant full-articles by A.K. and E.A.D.P., independently. For the update search, A.K. and R.T. assessed full-articles, independently. Disagreements were resolved by consensus and discussed with C.J.E. The inter-rater reliability between the first author A.K. and E.A.D.P. and between A.K. and R.T. were assessed with a percentage of agreement. The selection process was presented in a PRISMA 2020 flow diagram (Fig. 1) [20]. Data were extracted by A.K. The PRISMA guideline [20] informed the data extraction detailing trial eligibility criteria, target population, impact on clinical outcome, and stated limitations, study design, study aim, including intervention, participant eligibility criteria, participant characteristics, screening to recruitment rate and main outcomes.

### Quality appraisal of included studies

The author A.K. assessed the risk of bias in all included RCTs, as described in the Cochrane Handbook for Systematic Reviews of Interventions [33]. A revised Cochrane risk-of-bias tool for randomized trials is composed of the following five domains of bias: risk of bias arising from the randomization process; risk of bias due to deviations from intended interventions; risk of bias due to missing outcome data; risk of bias in measurement of the outcome; and bias in selection of the reported result. The summary judgements of the level of risk of bias for each domain are presented in Table 1. We used the robvis which is a visualization tool for tabulating a table of risk of bias and categorised them as, ‘low risk’, ‘high risk’, ‘some concerns’, or ‘no information’ [34].

**Table 1:** Characteristics of the included studies

Author (year), Country	Study design, Study aim (including the intervention)	Study setting	Mean age (SD/range), Gender (female %), Diagnosis	Primary outcome	Effect on primary outcome	Risk of bias
Ahronheim [35] (2000), US	Parallel RCT, To determine if a palliative care approach could be implemented for patients with advanced dementia and if this approach could enhance patient comfort	Hospital Inpatients	I/C = 83.9 (range 63–99)/85.6 (72–100), I/C = 77.1/86.3%, Dementia	･Mean number of hospitalizations ･Average length of stay, mortality	No significant difference	**High**
Bekelman [36] (2018), US	Parallel RCT, To determine whether a symptom and psychosocial collaborative care intervention improves heart failure specific health status, depression, and symptom burden in patients with heart failure	Hospital Out patients	65.5 (11.4), 21%, CHF	･Patient-reported HF-specific health status	Not significant improvement	**Low**
Bassi [37] (2021), Italy	Pilot RCT, To determine the feasibility and efficacy of a multidisciplinary palliative care approach to relieve patients’ symptoms and QOL	Hospital Out patients	I/C = 74.4(8.6)/77.4(6.9) I/C = 24/24% Advanced ILD	･Symptoms: dyspnoea, cough, depression (CES-D) ･Perceived QOL	Borg scale's and CES-D scale's values remained stable in the intervention group, while they both deteriorated in the control group (*P* < .05)	**High**
Gade [38] (2008), US	Parallel RCT, To measure the impact of an interdisciplinary palliative care service on patient satisfaction, clinical outcomes, and cost of care for 6 months posthospital discharge	Hospital Inpatients	I/C = 73.6(12.6) /73.1 (13.2), I/C = 59%/51%, Cancer, CHF	･Symptom control ･Levels of emotional/spiritual support ･Patient satisfaction	Higher mean satisfaction with hospital care and providers (*p* > 0.001)	**High**
Hanson [39] (2019), US	Pilot RCT, To develop a best-practice model of palliative care consultation for advanced dementia triggered by hospital admission for serious acute illness	Hospital Inpatient and Out Patients	I/C = 83.0(8.8)/84.7(8.7), I/C = 67/47% Advanced dementia	･60-day hospital or emergency department visits	No significant difference	**High**
Helgeson [40] (2022), US	Parallel RCT To investigate whether earlier palliative care medicine consultation in the ICU will result in decreased length of stay in the ICU and hospital, as well as, increased patient and family satisfaction	Hospital ICU	I/C = 71(range30-94)/71 (41–84) I/C = 40/41% ICU patients	･Patient satisfaction	The median satisfaction score (FS-ICU 24) was 23 points higher for the patients in the intervention group (*P* < .001)	**High**
Janssen [41] (2020) US	Pilot RCT To evaluate the effects of adding a palliative care intervention for patients with IPF to current standard of care	Hospital Out patients	I/C = 72.7(8)/69.5(7.2) I/C = 0/18% IPF	･Respiratory QOL ･Anxiety ･Depression	No significant difference	**High**
Kluger [42] (2020), US	Parallel RCT, To determine if outpatient palliative care is associated with improvements in patient outcomes compared with standard care	Hospital Out patients	I/C = 69.5(8.3)/70.7(8.0) I/C = 38.7/32.7% PD and related disorders	･QOL (QOL-AD) ･Caregiver burden	Significant difference in QOL (treatment effect estimate 1.87; 95% CI 0.47–3.27; *P* = .009)	**High**
O'Donnell [43] (2018), US	Pilot RCT, To determine if early initiation of social worker–aided palliative care would improve outcomes and influence care plans for high-risk patients discharged after HF hospitalization	Hospital Inpatients	I/C = 74.7(11.2) /69.2(10.2), I/C = 46.1/37.5%, Advanced HF	･Percentage of patients with physician-level documentation of advanced care preferences	Not significant improvement	**High**
O'Riordan [44] (2019), US	Pilot RCT, To determine if an interdisciplinary palliative care provided concurrently with standard cardiology care improves outcomes	Hospital Inpatients	I/C = 71(18)/59(19), I/C = 69/28%, HF	･Depression	No significant difference	**High**
Rogers [45] (2017), US	Parallel RCT, To investigate whether an interdisciplinary palliative care intervention in addition to evidence-based HF care improves certain outcomes	Hospital Inpatients	I/C = 71.9 (12.4) /69.8 (13.4), I/C = 44%/50.7%, Advanced HF	･HF-specific QOL (KCCQ) ･General and palliative care-specific, health-related QOL (FACIT-Pal)	Clinically significant incremental improvement in KCCQ and FACIT-Pal scores from randomization to 6 months	**High**
Schunk [46] (2021), Germany	Fast track RCT To evaluate the effectiveness of a multi-professional breathlessness service in patients with advanced and chronic diseases	Hospital Out patients	I/C = 71.9(8.9)/70.7(8.3) I/C = 52.2/49.5% Advanced life-limiting and progressive disease	･Patients’ mastery of breathlessness (CRQ) ･QOL ･Symptom	Significant improvement in CRQ Mastery of 0.367 [95% CI: 0.065; 0.669] score units for the early intervention group	**Some concerns**
Sidebottom [47] (2015), US	Parallel RCT, To assess if inpatient palliative care for HF patients is associated with improvements in symptom burden, depressive symptoms, QOL, or differential use of services	Hospital Inpatients	I/C = 76.0 (11.9) /70.9 (13.6), I/C = 52.6%/42.2%.cute HF	･Symptom burden ･Depressive symptoms ･QOL	Larger improvement on all three outcomes in the intervention group after adjustment for age, gender, and marital status	**High**
Aiken [48] (2006), US	Parallel RCT, To document outcomes of an RCT of the home-based palliative care and coordinated care/case management for seriously chronically ill individuals who simultaneously received active treatment	Home	I/C = 68(14)/70(13), I/C = 58/70%,HF, COPD	･Self-management of illness ･Preparation for end of life ･Physical/mental functioning ･Service use	Significant higher scores on the SF-36™ Physical functioning score at the 9-month point. (p < 0.05)	**High**
Bajwah [49] (2015), UK	Phase2: fast-track RCT, To obtain preliminary information on the impact of a case conference intervention delivered in the home on palliative care concerns of patients and their carers, and to evaluate feasibility and acceptability	Home	F/W = 67.1(10.9) /70.6 (10.3),F/W = 23/33%,ILD (IPF, NSIP)	･Palliative Care Outcome Scale (POS) (a measure of symptoms and concerns)	Significantly greater reduction in total POS between baseline and week 4 for the fast-track group. (ES: − 0.7; 95% CI − 1.2- − 0.1)	**High**
Brännström [50] (2014), Sweden	Parallel RCT, To evaluate the effects of the Person-centred and integrated CHF and palliative home care intervention on symptom burden, QOL, and functional classes compared with usual care	Home	I/C = 81.9(7.2) /76.6 (10.2),I/C = 27.8%/30.6%,HF	･Symptom burden (ESAS) ･Health-related QOL(EQ-5D) ･Functional classes	Improvement in total symptom score and HRQQL (*p* < 0.05)	**High**
Brumley [51] (2007), US	Parallel RCT, To determine whether an in-home palliative care intervention for terminally ill patients can improve patient satisfaction, reduce medical care costs, and increase the proportion of patients dying at home	Home	74 (12.0), 49%, Cancer, CHF, COPD	･Satisfaction with care ･Use of medical services ･Place of death	Greater improvement in satisfaction with care at 30, 90-day (OR53.37, 95%CI:1.42–8.10) (OR53.37, 95%CI:0.65–4.96)	**High**
Eggers [52] (2018), Germany	Parallel RCT, To identify if an integrated model of care for PD patients has access to PD patients at the end of life	Home	I/C = 69.8 (8.4) /69.9 (7.8), I/C = 31/35%, PD	･QOL (PDQ-39)	QOL significantly improved in the intervention group over a 6-month period. (2.2 points (95%CI: − 4.4—0.1); *p* = 0.044)	**High**
Evans [53] (2021), UK	Parallel RCT To evaluate the impact of the short-term integrated palliative and supportive care intervention for older people living with chronic noncancer conditions and frailty on certain outcomes	Home, care home	I/C = 85.3(6.4)/86.0(5.7) I/C = 50/46.2% Chronic noncancer conditions and frailty	･Symptoms (IPOS 3-day version)	The intervention reduced symptom distress (mean difference -1.20; 95% CI -2.37 to -0.027; omega squared = 0.071)	**Low**
Farquhar [54] (2009), UK	Phase2: fast-track RCT, To test the feasibility of single-blinding in a fast-track pragmatic RCT of BIS versus standard care for patients with a different non-malignant disease (COPD) and their informal carers	Home	Median 69 (range53-80), 39%, COPD/COAD	･Distress due to breathlessness measured using a VAS (0–10)	Not stated	**Low**
Farquhar [55] (2016), UK	Fast-track RCT, To establish the effectiveness and cost effectiveness of BIS in advanced non-malignant conditions	Home	F/W = 72.3 (10.6) /72.2 (9.4) F/W = 36/42%, Non-malignant disease	･Distress due to breathlessness measured using an NRS (0–10)	Non-significant greater reduction (–0.24, 95% CI: –1.30–0.82)	**Low**
Gao [56] (2020), UK	Parallel RCT, To determine the effectiveness of a short-term integrated palliative care intervention for people with long-term neurological conditions	Home	I/C = 67.3(10.9)/66.4(12.6) I/C = 51.1/46.6% Advanced LTNCs	･Symptoms (IPOS for neurological conditions)	No significant difference	**Some concerns**
Higginson [57] (2014), UK	Parallel group fast-track RCT, To assess the effectiveness of early palliative care integrated with respiratory services for patients with advanced disease and refractory breathlessness	Home	I/C = 66 (11)/68 (11), I/C = 47/37%, COPD, cancer, ILD, CHF	･Patient-reported breathlessness mastery (CRQ) ･QOL (CRQ)	Mastery in the BSS group (MD: 0.58, 95% CI: 0.01–1.15, *p* = 0.048; ES 0.44) and total QOL improved	**Low**
Janssen [58] (2019), Switzerland	Pilot RCT To assess the effectiveness of the introduction of early specialized palliative care on hospital, ICU and emergency admissions of patients with severe and very severe COPD	Home	I/C = 70.8(8.4)/71.3(8.1) I/C = 46.2/60.9% Severe COPD	･Length of stay hospital, ICU and emergency admissions	No significant difference	**High**
Scheerens [59] (2020), Belgium	Pilot RCT, To test feasibility, acceptability, and preliminary effectiveness of early integrated palliative home care for end-stage COPD	Home	I/C = 67.5(8.4)/67.4(7.9) I/C = 45/42.1% End-Stage COPD	Not defined ･Hospitalizations ･HRQOL etc	No overall intervention effect for the outcomes	**High**
Ng [60] (2018), Hong Kong	Parallel RCT, To examine the effect of a home-based palliative heart failure program on QOL, symptoms burden, functional status, patient satisfaction, and caregiver burden among patients with ESHF	Home	I/C = 78.3 (16.8) /78.4 (10.0), I/C = 56.1%/39% End-stage HF	･QOL (McGill QOL Questionnaire-Hong Kong)	Significant improvements in the physical (*p* = 0.011), psychological (*p* = 0.04), and existential (*p* = 0.027) domains	**High**
Wong [61] (2016), Hong Kong	Parallel RCT, To examine the effects of home-based transitional palliative care for patients with ESHF after hospital discharge	Home	I/C = 78.3 (16.8) /78.4 (10.0), I/C = 56.1%/39%, Advanced HF	･Count of readmission	Significantly lower readmission rate at 12 weeks. (intervention 33.6% vs control 61.0% χ2 = 6.8, *p* = 0.009)	**High**

***Abbreviations:**** BIS* breathlessness intervention service*, BSS* breathlessness support service*, CES-D* the Center for Epidemiologic Studies Depression Scale*, CHF* chronic heart failure*, COAD* chronic obstructive airway disease*, COPD* chronic obstructive pulmonary disease*, CRQ* the chronic respiratory disease questionnaire*, EQ-5D* EuroQol 5-dimensions*, ES* effect size*, ESAS* the Edmonton Symptom Assessment System*, ESHF* end-stage heart failure*, F/W* fast track/waiting list*, FACIT-Pal* the functional assessment of chronic illness therapy-palliative*, FS-ICU* the Family Satisfaction with the ICU Survey*, HRQOL* health-related quality of life*, HF* heart failure*, I/C* intervention/control, *ICU* intensive care unit*, ILD* interstitial lung disease*, IPF* idiopathic pulmonary fibrosis*, IPOS* the Integrated Palliative care Outcome Scale*, KCCQ* the Kansas city cardiomyopathy questionnaire*, LTNC* long-term neurological conditions, *MD* mean difference*, NRS* numerical rating scale*, NSIP* non-specific interstitial pneumonia*, OR* Odds ratio*, PD* Parkinson's disease*, PDQ* the Parkinson's disease questionnaire*, POS* the Palliative care Outcome Scale*, QoL-AD* the Quality of Life in Alzheimer’s disease*, RCT* randomised controlled trial, *SD* standard deviation*, SF36* the 36-Item Short Form Health Survey*, SGRQ* the St. George’s Respiratory Questionnaire*, VAS* visual analogue scale

### Data analysis and synthesis

Descriptive analysis and narrative synthesis provided descriptions of the patterns and appraised the applicability of included trial eligibility criteria to identify patients likely to benefit from receiving palliative care. We sought to identify and collate eligibility criteria and appraise what patterns of eligibility criteria were successful in terms of recruitment, attrition, attaining sample size, and effect on the primary outcome. Eligibility criteria were summarized by frequency counts of domains and synthesized by intended sample size, attrition rate, causes of attrition, and limitation that reflected on the eligibility.

To assess the recruitment, we appraised whether: 1) the study recruited the relevant population to answer the study aim, 2) the actual sample size was larger than the intended sample size determined by sample size calculation, and 3) the explanation for revision of the target sample size was given.

We assessed the attrition rate in accordance with the implementation of sample size estimation, cause of attrition, and anticipated attrition rate. Rates of attrition in the included trials were assessed to explore levels of attrition and if high attrition, to consider the appropriateness of the trial eligibility criteria to identify patients for palliative care (or not). To describe causes of attrition, we used the MORECare classification of attrition to describe causes of attrition: attrition due to death (ADD), attrition due to illness (ADI), and attrition at random (AaR) [62, 63]. While there is no standardised level of loss to follow-up which attrition related bias was identified as a problem, Schulz and Grimes noted that the readers should be concerned about the possibility of bias when the attrition rate was 20% or greater [64]. However, the weighted average attrition across palliative care trials involving adults with serious illness and increasing nearness to end of life in a systematic review was 29% [63]. For example, a review of interventional palliative oncology trials stated that the attrition rate was 26% for the primary endpoint and 44% for the end of the study [65]. Therefore, we considered high attrition rate when the rate was more than 25%. Although dropouts due to symptom progression or death were not considered as protocol failures in palliative care trials, we sought to appraise what patterns of eligibility criteria were successful in terms of recruitment and the effect on the primary outcome.

We identified for each trial the level of statistically difference on the primary outcome between the intervention and control groups and the effect size. We then explored the respective eligibility criteria and target population to map and identify criteria associated with effect on the primary patient outcome. This intended to explore further the appropriateness (or not) of the patient eligibility criteria used. Because the timing of outcome measurement could influence the attrition rate and the effect on the primary outcome, we also considered the impact of length of intervention and time points of data collection.

## Results

### Study selection

The electronic search strategy identified total 9,584 papers (6,720 in 2018 and 2,850 in the update search). An additional 21 papers were identified by hand searching and citation tracking. After removing duplicates, 7,134 studies were screened at title and abstract, and 67 were assessed as full-text articles. 27 met eligibility (20 studies identified by electronic searches and seven from hand search) (see Fig. 1) [35–61]. All included studies were written in English. The reasons for study exclusion are reported in Fig. 1 [66–103]. The inter-rater agreement between independent reviewers of full text screening were 89% in the initial search and 86% for the update search.

### Quality appraisal

Only five studies were considered low risks of bias [36, 37, 50, 52, 53]. 20 studies had high risks of bias, mainly due to incomplete outcome data. Two study were assessed at some concerns because of unstated data and lack of information [46, 56]. The risk of bias plots are presented in Additional files 3 and 4. The average attrition rate was 23%, ranging from 0 to 52%. The major cause of attrition was death. Eleven papers stated that small sample size was one of the limitations. What we valued more than attrition rate when assessing incomplete outcome data was advance estimations of attrition, descriptions of reasons for missing data, and whether they integrated these into sample size calculation.

### Study characteristics

This review included 27 RCTs written in English. Most studies were conducted in US. This is important to understand the context of the work and the applicability of the proposed triggers for which settings. The first study was conducted in US in 2000 [35]. (Table 1).

### Study design

We included 18 phase III RCTs and nine feasibility trials. The 15 parallel RCTs compared the palliative care intervention with usual care. Five studies used fast-track design and compared a fast-track group with a waiting list group. Higginson et al. [57] used a parallel group fast-track trial design. Finally, five studies used a mixed methods trial design [49, 50, 53, 54, 57].

### Participants

The studies included 3,663 participants ranging from 14 to 517 per study [54, 55]. The mean age ranged from 65.5 years with heart failure [36] to 85.7 years with chronic noncancer conditions and frailty [53]. The female percentage ranged from 9.1% [41] to 81.8% [35]. Eleven studies described the ethnicity of the participants; the majority of participants were White, followed by African Americans.

Of the included 27 papers, eight were conducted with patients with heart failure (HF) [36, 43–45, 47, 50, 60, 61]. Six included participants with respiratory disease, two with COPD [58, 59], two with interstitial lung disease (ILD) [37, 49], one with idiopathic pulmonary fibrosis (IPF) [41], and one with COPD/chronic obstructive airway disease (COAD) [54]. Other diagnoses were neurological diseases, two with Parkinson’s disease (PD) [42, 52], one with dementia [35], and one stated long-term neurological conditions. Four studies [38, 48, 51, 57] included multiple diseases, three included both cancer and noncancer conditions [38, 51, 57], and one included both CHF and COPD [48]. Three studies stated general chronic/advanced noncancer populations [46, 53, 55], and one included intensive care unit (ICU) populations [40].

### Intervention and control

Eleven different models of home palliative care in 14 studies were identified [48–61]. All eleven models were composed of multi-components of palliative care intervention, such as symptom management, self-management education of disease, end-of-life discussions, case conferences, documentation, regular home visits, or a telephone hotline. Seven studies [35, 38, 40, 43–45, 47] provided inpatient care and six [36, 37, 39, 41, 42, 46] implemented outpatient palliative care services. Inpatient palliative care services in seven studies were developed based on the standard referral process of the hospital palliative care team or developed for the trial. As for control group, usual care differed across studies due to the wide variety of health systems and local service provisions. Several studies followed national or government guidelines.

### Primary outcomes

20 studies [36–38, 41, 42, 44–50, 52–57, 59, 60] set quality of life (QOL) or symptom burden as a primary outcome, with marked heterogeneity in the measurements used. (Table 1). Eight used disease-specific QOL measurements, two used HF-specific QOL measures; The Kansas City Cardiomyopathy Questionnaire (KCCQ), four used respiratory diseases specific measures; a QOL domain in the Chronic Respiratory Disease Questionnaire (CRQ), the St. George’s Respiratory Questionnaire (SGRQ), or Maugeri Respiratory Questionnaire (MRQr), and two used measures for neurological conditions; the 39-Item Parkinson's Disease Questionnaire (PDQ-39) or the Quality of Life in Alzheimer’s Disease (QoL-AD). Other primary outcomes were medical service use, documentation of care preferences, and patient satisfaction.

### Summary of eligibility criteria of included studies

Six main domains for eligibility criteria were identified, including diagnostic criteria (*n* = 26 out of 27 included studies, 96%), medical history-based (*n* = 15, 56%), symptoms (physical and psychological) (*n* = 14, 52%), prognostic criteria (*n* = 9, 33%), functional status (n = 8, 30%]), QOL (*n* = 2 [7%]), and other criteria (*n* = 4, 15%). We categorised these domains into three major criteria themes: needs-based, time-based, and medical history-based.

In Table 2, we used the initial letter of each domain to show which category the eligibility trials were categorised. The letter D stands for ‘Diagnostic Criteria’, P for ‘Prognostic’, S for ‘Symptoms’, Q for ‘QOL’, F for ‘Functional Status’, M for ‘Medical History and Treatment’, and O for ‘Other’. The number of domains was calculated by adding the number of domains used in the trial as eligibility criteria. Since some standardised measures covered several domains, we analysed the domain in the measurements and counted the number of domains. For example, Bekelman et al. [36] used a score of HF-specific health status (KCCQ) to assess eligibility. As KCCQ is a reliable and valid measure of symptoms, functional status, and QOL, the number of domains counted was three [104]. Table 3 gives an overview of the different criteria and use by respective disease groups. Table 4 gives a systematization of the eligibility criteria which shows major categories for referral criteria.

**Table 2:** Comparison of trial eligibility criteria and rates and cause of attrition

**Author (year)**	**Trial eligibility criteria** **domains** (**D:** Diagnostic criteria, **P:** Prognostic criteria, **S:** Symptoms, **Q:** QOL, **F:** Functional status, **M:** Medical history/treatment, **O:** Others)	**N of domains**	**Intended sample size**	**Sample / sample size (attrition %)**	**Causes of** **attrition** (causes/N of loss)	**Limitation that reflected on the eligibility**
Ahronheim [35] (2000)	**M**: Hospitalization for acute illness **D, F**: FAST 6d or greater (Dementia)	3	Not stated	99/99 (0%)	NA	Sample was small. (Patients should be identified prior to the acute hospitalization)
Aiken [48] (2006)	**D**: NYHA IIIB – IV (HF), Oxygen saturations, pO2, oxygen requirements (COPD) **P**: Expert judgment based on available prognostic data **S**: Fatigue, palpitation, dyspnoea, or angina due with any activity **M**: Recent exacerbation (treatment in a hospital within the 3-month prior to enrolment)	4	Post-hoc calculation	112/192 at 3-month (42%), 92/192 at 6-month (52%)	Medical causes (death, hospice, skilled nursing facilities)	The prognostication criteria were limited. One third of all participants died or transferred to hospice in the first 3 months
Bajwah [49] (2015)	**D**: High resolution CT or composite physiologic index scores (ILD)	1	Phase2: 52	35/53 (34%)	Died (7/18), no return of questionnaire but contactable (7/18)	The criteria for excluded patients were not recorded which may have provided valuable information
Bekelman [36] (2018)	**S**: At least one symptom (fatigue, shortness of breath, pain, depression) **F, Q**: KCCQ, **M**: Diuretic dosing, LVEF, BNP, NT-pro BNP	4	312	248/317 (22%)	Deceased (8/69) Withdraw (8/69)	The missing patient-reported data is similar to other studies of seriously ill populations
Bassi [37] (2021)	**D**: ILD defined by a HRCT (with traction bronchiectasis and/or honeycombing) **P**: evidence of advanced disease: GAP index at least 3, PaO2 ≤ 60 mmHg at room air, a decline in FVC ≥ 10% in the previous 6 months	2	50	33/50 (34%)	Death (14/17) Refused to continue (3/17)	Enrolled patients were in a late phase of the disease, where palliative interventions may have limited opportunity for effectiveness
Brännström [50] (2014)	**D**: NYHA III − IV (CHF) and at least one of the following: **P**: < 1 year (no criteria), **S**: Cardiac cachexia—weight loss, **Q**: QOL (VAS) **M**: Hospitalization of worsening HF that resolved with the IV, continual IV support	5	72	60/72 (17%)	Died (12/12)	Patients with a high number of severe co-morbidities lead small sample
Brumley [51] (2007)	**D**: CHF, COPD, cancer. (severity was assessed by PPS), **P**: Surprise Question **F**: PPS, **M**: Visited ED or hospital at least once within the previous year of enrolment	4	300	297/310 (4%)	Died before intervention (8/13), Withdraw (5/13)	Not stated the eligibility criteria
Eggers [52] (2018)	**D**: Parkinson’s disease (no criteria)	1	150	107/150 (29%)	Withdraw (18/43), loss of contact (15/43)	Exclusion criteria (dementia or severe depression) are a serious limitation for the inclusion of late-stage PD
Evans [53] (2021)	**D**: Non-malignant chronic conditions, **F**: Clinical Frailty Scale sore of ≥ 4 **S, F**: ≥ 2 symptoms or concerns, including end-of-life issues, progressive illness/frailty, and/or complex needs **M**: Increasing health service use	4	5	47/50 (6%)	Deceased (1/3) New cancer diagnosis (1/3) Patient unwell (1/3)	Not stated a limitation of the eligibility criteria
Farquhar [54] (2009)	**D**: COPD/COAD (no criteria) **S**: Breathlessness in spite of optimisation of underlying illness **O**: Patients who might benefit from a self-management programme	3	Phase 2: Maximum of 28	13/14 (7%)	Died (1/1)	Not stated the eligibility criteria
Farquhar [55] (2016)	**D**: Non-malignant (no criteria) **S, O**: Same as the phase 2 trial	3	60	72/87 (17%)	Died (2/15)	Not stated the eligibility criteria
Gade [38] (2008)	**D**: Life-limiting diagnosis (no criteria) **P**: Surprise Question	2	550	512/517 (1%)	Withdrew prior to the intervention	Not stated the eligibility criteria
Gao [56] (2020)	**D**: MS (EDSS score ≥ 7.5), all stages of MND, IPD (Hoehn and Yahr stages 4–5), progressive supranuclear palsy (Hoehn and Yahr stages 3–5) and multiple system atrophy (Hoehn and Yahr stages 3–5) **S**: An unresolved symptom; cognitive problems or complex psychological issues; communication or information problems or complex social need	2	35	327/350 (7%)	Died (8/23) Withdraw (15/23)	Sample was largely composed of patients with MS and IPD who tend to have a longer disease course
Hanson [39] (2019)	**D**: Dementia stage 5 to 7 on the GDS **M**: Acute illness hospitalization	2	120	57/62 (8%)	Lost to follow up (4/5) Withdraw (1/5)	Many persons could not be enrolled due to short hospital stays and caregiver stresses
Helgeson [40] (2022)	**D**: End-stage organ disease,** P**: Age ≥ 80 years, APACHE II ≥ 14, SOFA ≥ 9; **F**: Pre-existing functional dependency (admitted from an acute living facility, skilled nursing facility, or long-term acute care facility), late-stage dementia (bed-bound, nonverbal, incontinent, or unable to self-nourish), **O**: MICU perceived need **M**: Consideration to place a permanent feeding tube or tracheostomy; recurrent ICU admissions in the past year; post-cardiac arrest	5	300	91/91 (0%)	NA	The criteria have a potential source of bias that the sicker patients would screen positive
Higginson [57] (2014)	**D**: Cancer, COPD, CHF, ILD, MND (no criteria) **S**: Refractory breathlessness (MRC scale) **O**: Willing to engage with BSS	3	110	82/105 (22%)	Died (4/23), withdrew (5/23), illness (8/23), unable to contact	Eligibility criteria prevented extrapolation of study results to patients in the last month of life
Janssen [58] (2019)	**D**: COPD in GOLD (FEV1/FVC < 70%) stage III or IV (FEV1 < 50% predicted) **M**: Long-term oxygen therapy and/or home mechanical ventilation and/or one or more hospital admissions in the previous year for an acute exacerbation	2	180	41/51 (20%)	Died (9/10) Declined (1/10)	Did not reach the target number of cases because of cognitive impairment, comorbidities, or end-of-life
Janssen [41] (2020)	**D**: IPF as diagnosed by chest CT or lung biopsy, and documented by a pulmonologist in the patient's medical record	1	Not stated	18/22 (18%)	Lung transplant (1/4) Died (1/4), Lost to follow-up (1/4) Hospice (1/4)	Logistical issues such as lack of interest in extra visits. Some patients felt the intervention was unnecessary at their stage in the disease
Kluger [42] (2020)	**D**: Diagnosis of probable PD or another PDRD (multiple system atrophy, corticobasal degeneration, progressive supranuclear palsy, or Lewy body dementia), **S, F**: Moderate to high PC needs based on the PC-NAT modified for PD	3	300	182/210 (13%)	Death (7/28) Withdraw (19/28) Contact to lost (1/28)	The study had broad inclusion criteria, but more focused recruitment could improve certain outcomes
O'Donnell [43] (2018)	**D**: NYHA II-IV (HF) **M**: Hospitalization with high-risk features	2	Not stated	31/50 (38%)	Died (19/19)	Small sample size
O'Riordan [44] (2019)	**D**: HF as primary diagnosis (NYHA Class II-IV) **M**: symptomatic/active HF in current hospitalization or within prior six months	2	84	30/39 (23%)	Dropped out (5/10), Not eligible (3/10) Died (2/10)	Many patients improved significantly by discharge and may not have needed an intensive, six-month palliative care intervention
Rogers [45] (2017)	**D**: HF with at least 1 sign of volume overload, **P**: ESCAPE risk score **S**: Dyspnoea at rest or minimal exertion, **M**: Hospitalization for acute HF	4	200 → 150	84/150 (44%)	Died (43/66)	High mortality and loss of follow-up reflect the difficulty of retaining seriously ill patients
Scheerens [59] (2020)	**D**: GOLD III and ≥ 2 or GOLD IV (COPD) and one or more of the following criteria: **S**: MRC Scale Dyspnea 4, NYHA III, BMI ≤ 18, **S, F**: CAT scale ≥ 25 **M**: Oxygen dependent, three or more hospitalizations for COPD in the past three years, intubation/noninvasive ventilation in the past year	4	40	25/ 39 (36%)	Died (6/14) Refused (6/14) Ended period (1/14) Too ill (1/4)	Although some GPs criticized the intervention being given too early, the criteria may be appropriate given the emphasis on early palliative care
Schunk [46] (2021)	**D:** Advanced life-limiting and progressive disease **S**: Breathlessness on exertion or at rest despite treatment of the underlying condition **F**: Capable to participate physiotherapy and self-management programs	3	160	143/183 (22%)	Withdrawal (23/40) Death (10/40) Medical reason (6/40) Lost to f/u (1/40)	Higher proportion of lost to follow-up were possibly caused by the burden relating to participating in the multi-component intervention
Sidebottom [47] (2015)	**D**: Acute HF (reports from the electronic record)	1	500	143/232 (38%)	Not completed survey, reason unknown (68/89), Died (19/89)	Losses to follow-up
Ng [60] (2018)	**D, P, S**: Same as Wong et al. [61]** M**: Repeated hospitalization (> two in last six months)	4	78	45/84 (46%)	Death, too ill, refusal (29/39)	Small sample due to the subjects being too weak or cognitively impaired
Wong [61] (2016)	Two of the following identified as ESHF by the Prognostic Indicator Guidance **D**: NYHA III-IV (HF) **P**: Surprise Question, **S**: Existence of physical/psychological symptoms despite optimal tolerated therapy **M**: Repeated hospital admissions with symptoms of HF (three within 1 year)	4	Not stated	68/84 (19%)	Discontinued interven tion (e.g., die) (14/16)	The loss of follow-up was high due to mainly death and deterioration

***Abbreviations:**** APACHE* the Acute Physiology And Chronic Health Evaluation score*, BMI* body mass index, *BNP* brain natriuretic peptide, *BSS* breathlessness support service*, CAT* COPD Assessment Test, *CHF* congestive heart failure*, COAD* chronic obstructive airways disease, *COPD* chronic obstructive pulmonary disease, *CT* computed tomography, *ED* emergency department*, EDSS* the Expanded Disability Status Scale, *ESCAPE risk score* the Evaluation Study of Congestive Heart Failure and Pulmonary Artery Catheterization Effectiveness risk score*, ESHF* end-stage heart failure*, f/u* follow/up*, FAST* functional Assessment Staging Tool*, GDS* the Global Deterioration Scale*, GOLD* the Global Initiative for Chronic Obstructive Lung Disease*, GP* general practitioner*, HF* heart failure*, HRCT* high-resolution chest CT*, ILD* interstitial lung disease*, IV* intravenous*, IPD* Idiopathic Parkinson's Disease*, JVP* jugular venous pressure*, KCCQ* the Kansas City Cardiomyopathy Questionnaire*, LVEF* left ventricular ejection fraction*, MICU* medical intensive care uni*t, MND* motor neurone disease*, MRC* Medical Research Council*, N* number*, MS* Multiple sclerosis*, NT-pro BNP* N-terminal prohormone level of BNP*, NYHA* New York Heart Association*, PC-NAT* the Palliative Care Needs Assessment Tool*, PD* Parkinson’s disease*, PDRD* Parkinson’s disease related disorders*, PPS* the Palliative Performance Scale*, QOL* quality of life*, SOFA* The Sequential Organ Failure Assessment, *VAS* visual analogue scale

**Table 3:** Summary of trial eligibility criteria by disease type

Diagnostic criteria	HF	NYHA II-IV [43, 44], NYHA III-IV [50, 60, 61], NYHA IIIB or IV [51], NYHA III [59]
	COPD	Oxygen saturations of less than 88%, or baseline pO_2_ less than 55, and to be on continuous oxygen [48] GOLD stage III or IV [40, 58, 59] Oxygen dependent [59]
	IPF, ILD	End stage IPF as judged by either high resolution CT or composite physiologic index scores > 50, clinical status, oxygen requirements, severe PH for too unwell patients [49] IPF diagnosed by chest CT or lung biopsy [41] ILD defined by a HRCT (with traction bronchiectasis and/or honeycombing) [37]
	Dementia	FAST 6d or greater [35] GDS stage 5, 6 or 7 [39]
	Neurological conditions	MS (EDSS score ≥ 7.5), MND (All stages), IPD (Hoehn and Yahr stages 4–5), Progressive supranuclear palsy / Multiple system atrophy (Hoehn and Yahr stages 3–5) [56]
	Other (no criteria)	Hospitalized with life-limiting diagnosis [38], End-stage organ failure [40] Non-malignant disease [55], Non-malignant chronic conditions [53] Advanced disease: cancer, COPD, CHF, ILD, MND [57], COPD/COAD (no criteria) [54] Acute HF (reports from the electronic health record) [47] HF as primary diagnosis [44], Parkinson’s disease (no criteria) [36] Probable PD or another PDRD (multiple system atrophy, corticobasal degeneration, progressive supranuclear palsy, or Lewy body dementia) [42]
Prognostic criteria	General	Surprise Question: Life expectancy of 12 months or less.“Would I be surprised if this patient died in the next 12 months?” [38, 51, 60, 61] Age ≥ 80 years, APACHE II ≥ 14, SOFA ≥ 9 [40]
	HF	ESCAPE risk score ≥ 4 (indicating > 50% predicted 6-month mortality) [45]
		ESHF by the Prognostic Indicator Guidance (Hospitalization with at least one of the following high-risk features) [60, 61] 1. Prior hospitalization for HF within 1 year, 2. Age ≥ 80 years, 3. CKD (estimated GFR ≥ ≤ 45 mL/min/m2 177), 4. Systolic Blood Pressure ≤ 100 mm Hg 5. Serum sodium ≤ 130 mEq/L, 6. Cardiogenic Shock (Cardiac Index ≤ 2.0) 7. Serious Non-Cardiovascular Illness (e.g. advanced stage cancer, COPD, or the like)
	ILD	GAP index at least 3, PaO2 ≤ 60 mmHg at room air, a decline in FVC ≥ 10% in the previous 6 months [37]
	Other (no criteria)	Live for up to 2 years, based on expert judgment that drew on available prognostic data [48] Life expectancy of < 1 year [55]
Symptoms (physical /psycho social)	General	MRC dyspnoea scale score (refractory breathlessness) [57] ≥ 2 symptoms or concerns including end-of-life issues, like advance care planning and/or complex needs (i.e. multiple psychosocial or physical symptoms or concerns) [53] Breathlessness due to life-limiting disease despite treatment of the underlying condition [46]
	HF	Symptoms (fatigue, palpitation, dyspnoea, or angina) due with any activity [48, 62] Cardiac cachexia (involuntary non-oedematous weight loss ≥ 6% of total body weight within the preceding 6–12 months) [50] Existence of physical/psychological symptoms despite optimal tolerated therapy [60, 61] Dyspnoea at rest or minimal exertion plus at least 1 sign of volume overload (JVP > 10 cm, peripheral oedema, congestion on chest x-ray) [60] At least one symptom (fatigue, shortness of breath, pain, and/or depression) [36]
	COPD	Breathlessness in spite of optimisation of underlying illness [54, 55] CAT scale ≥ 25, MRC Scale Dyspnoea 4, NYHA III, BMI ≤ 18 [59]
	Neurological conditions	An unresolved symptom which had not responded to standard care, an unresolved other symptom, cognitive problems or complex psychological issues, communication or information problems or complex social need [56] Moderate to high PC needs based on the PC-NAT modified for PD [42]
Functional status	General	PPS 70% or less [51] Clinical Frailty Scale sore of ≥ 4 [53] Capable to participate physiotherapy and self-management programs [46] Pre-existing functional dependency (admitted from an acute living facility, skilled nursing facility, or long-term acute care facility) [40]
	HF	HF–specific health status (KCCQ score of ≤ 70) [36]
	COPD	CAT scale ≥ 25 [59]
	Dementia	FAST of 6d or greater [34, 35] Late-stage dementia (bed-bound, nonverbal, incontinent, or unable to self-nourish) [40]
	PD	Moderate to high PC needs based on the PC-NAT modified for PD [42]
QOL	General	QOL (VAS < 50) [50]
	HF	HF–specific health status (KCCQ score of ≤ 70) [36]
Medical history /treatment	General	Consideration to place a permanent feeding tube or tracheostomy, recurrent ICU admissions in the past year, post-cardiac arrest [40] Increasing health service use [53]
	HF	Repeated hospital admissions with symptoms of HF (three within 1 year [49]/ > 2 in last 6 months [60]) Symptomatic/active HF in current hospitalization or within prior six months [44] A hospitalized episode of worsening HF that resolved with the injection/infusion of diuretics or the addition of other HF treatment in the preceding 6 months despite being optimally treated [50] Need for frequent or continual IV support [50] Required diuretic dosing (furosemide ≥ 80 mg/d or equivalent), LVEF of 40% or less, BNP levels of 250 pg/mL or more, or NT-pro BNP of 1000 pg/mL or more [36] Hospitalization for acute HF [45], Hospitalization with high-risk features [43]
	HF/COPD	Recent exacerbation (treatment in an ED, urgent care facility, or hospital within the 3 months prior to enrolment) [48] Visited ED or hospital at least once within the previous year of enrolment [51]
	COPD	LTOT, home NIV, hospital admissions in the previous year for an acute exacerbation. [58] Three or more hospitalizations for COPD in the past three years, intubation in the past year, non-invasive ventilation in the past year [59]
	Dementia	Hospitalization for acute illness [35, 39]
Others	General	Patients who might benefit from a self-management programme [54, 55] Willing to engage with willing to engage with short-term home physiotherapy and occupational therapy [57] MICU perceived need [40]

***Abbreviations:**** APACHE* the Acute Physiology And Chronic Health Evaluation score, *BMI* body mass index, *BNP* brain natriuretic peptide, *CAT* COPD Assessment Test, *CHF* congestive heart failure, *CKD* chronic kidney disease, *COAD* chronic obstructive airways disease, *COPD* chronic obstructive pulmonary disease, *CT* computed tomography, *ED* emergency department, *EDSS* the Expanded Disability Status Scale, *ESCAPE risk score* the Evaluation Study of Congestive Heart Failure and Pulmonary Artery Catheterization Effectiveness risk score, *ESHF* end-stage heart failure, *FAST* functional Assessment Staging Tool, *GAP* the Gender-Age-Physiology index, *GDS* the Global Deterioration Scale, *GFR* glomerular filtration rate, *GOLD* the Global Initiative for Chronic Obstructive Lung Disease, *HF* heart failure, *HRCT* high-resolution chest CT, *ICU* intensive care unit, *ILD* interstitial lung disease, *IPD* Idiopathic Parkinson's Disease, *IPF* idiopathic pulmonary fibrosis, IV intravenous, *JVP* jugular venous pressure, *KCCQ* the Kansas City Cardiomyopathy Questionnaire, *LTOT* long-term oxygen therapy, *LVEF* left ventricular ejection fraction, *MICU* medical intensive care unit, *MND* motor neurone disease, *MRC* Medical Research Council, *MS* Multiple sclerosis NIV non-invasive ventilation*, NT-pro BNP* N-terminal prohormone level of BNP, *NYHA* New York Heart Association, *PC* palliative care, *PC-NAT* the Palliative Care Needs Assessment Tool, *PD* Parkinson’s disease, *PDRD* Parkinson's disease and related disorders, *PH* pulmonary hypertension, *PPS* the Palliative Performance Scale, *QOL* quality of life, *SOFA* The Sequential Organ Failure Assessment, *VAS* visual analogue scale

**Table 4:** Criteria for palliative care referral

Study, year [reference]	Time-based criteria		Needs-based criteria			Treatment	Others
	Diagnostic criteria	Prognostic criteria	Symptoms	Functional status	QOL	Medical history	
Ahronheim, 2000 [35]	+			+		+	
Aiken, 2006 [48]	+	+	+			+	
Bajwah, 2015 [49]	+						
Bekelman, 2018 [36]			+	+	+	+	
Bassi, 2021 [37]	+	+					
Brännström, 2014 [50]	+	+	+		+	+	
Brumley, 2007 [51]	+	+		+		+	
Eggers, 2018 [52]	+						
Evans, 2021 [53]	+		+	+		+	+
Farquhar, 2009 [54]	+		+				+
Farquhar, 2016 [55]	+		+				+
Gade, 2008 [38]	+	+					
Gao, 2020 [56]	+		+				
Hanson, 2019 [39]	+					+	
Helgeson, 2022 [40]	+	+		+		+	+
Higginson, 2014 [57]	+		+				+
Janssen, 2019 [58]	+					+	
Janssen, 2020 [41]	+						
Kluger, 2020 [42]	+		+	+			+
O'Donnell, 2018 [43]	+					+	
O'Riordan, 2019 [44]	+					+	
Rogers, 2017 [45]	+	+	+			+	
Scheerens, 2020 [59]	+		+	+		+	
Schunk, 2021 [46]	+		+	+			
Sidebottom, 2015 [47]	+						
Ng, 2018 [60]	+	+	+			+	
Wong, 2016 [61]	+	+	+			+	
**Total, *n* (%)**	**26 (96)**	**9 (33)**	**14 (52)**	**8 (30)**	**2 (7)**	**15 (55)**	**6 (22)**

***Note***: Plus signs indicate that study used the criterion; blank cells indicate that study did not use the criterion

***Abbreviation***: *QOL* Quality of life

### Need-based criteria

Focused on three main areas of symptoms, function, and quality of life, including:

### Symptoms

Around half of the 27 included studies set an existence of physical/psychological symptoms as eligibility criteria [36, 42, 45, 46, 48, 50, 53–57, 59–61]. As for physical symptoms, Aiken et al. [48] included HF or COPD patients suffering from fatigue, palpitation, dyspnoea, or angina with any activity. For participants with HF, Brännström et al. [50] checked the presence of cardiac cachexia with involuntary non-oedematous weight loss ≥ 6% of total body weight within the preceding 6–12 months, and Bekelman et al. [36] confirmed reporting at least one of the target symptoms of fatigue, shortness of breath, pain, and/or depression. Only six studies [36, 42, 53, 56, 60, 61] contained psychological symptoms as eligibility criteria. Four studies that provided breathlessness intervention/support service examined whether breathlessness existed in spite of optimisation of the underlying illness [46, 50, 54, 57]. Among them, Higginson et al. [57] used the Medical Research Council (MRC) dyspnoea scale to assess the degree of refractory breathlessness. Four studies [42, 53, 56, 59] conducted comprehensive screening of complex symptoms in palliative population.

### Functional status

Eight studies included criteria that assess functional or performance status in their eligibility [35, 36, 40, 42, 46, 51, 53, 59]. In trials of patients with HF or COPD, the Palliative Performance Scale (PPS) and KCCQ were employed to assess functional status alongside prognosis, symptoms, or QOL [36, 51]. Regarding neurological conditions, Ahronheim et al. [35] used the Functional Assessment Staging Test (FAST) for systematic examination of the functional changes occurring in patients with dementia. Helgeson et al. [40] considered admission of patients with dementia from nursing care facilities as a pre-existing functional dependency. The trial eligibility criteria of Kluger et al. [42] on Parkinson's disease contain the Palliative Care Needs Assessment Tool (PC-NAT) and their criteria were based on a broad range of potential palliative care needs rather than time-based criteria.

Evans et al. [53] assessed the existence of frailty with the clinical frailty scale score. Shunk et al. [46] set the capability to participate in physiotherapy as a functional criterion because of the nature of the intervention programme.

### Quality of life

Only two trials used QOL for trial eligibility [36, 50]. Bekelman et al. [36] used KCCQ in their trial as a measurement of the patient’s perception of their health status which includes how their heart failure impacts their QOL within a 2-week recall period. Brännström et al. [50] measured QOL using a Visual Analogue Scale (VAS). VAS is commonly used to rate subjective experiences [105].

### Time-based criteria

#### Diagnostic criteria

Diagnostic criteria were a set of signs and tests for use in routine clinical care to guide the care of individual patients. In the 12 studies that included HF, six studies [43, 44, 48, 50, 60, 61] used the New York Heart Association (NYHA) classification of II-IV [43, 44], or NYHA III-IV [47, 48, 50, 60]. Brumley et al. [51] included not only participants with HF, but also COPD and cancer, and used the Palliative Performance Scale to assess disease severity. Rogers et al. [45] measured signs of volume overload in accordance with the HF diagnosis. But, three HF studies [38, 47, 57] used no diagnostic criteria.

Similarly, diagnostic eligibility criteria were used in studies on lung disease and neurological conditions. Aiken et al. [48] in a study on COPD used measures of hypoxemia, oxygen saturation, pO2, and oxygen requirements. Three studies [37, 41, 49] on ILD or IPF used high-resolution computed tomography of lung or a composite physiologic index. Janssen et al. (2019) [58] and Scheerens et al. [59] used the Global Initiative for Chronic Obstructive Lung Disease (GOLD) system to categorize airflow limitation into stages of COPD.

Regarding neurological conditions, Gao et al. [56] employed the Hoehn and Yahr scale and the Expanded Disability Status Scale (EDSS) to describe the progression of each neurological disease. Hanson et al. [39] used the Global Deterioration Scale (GDS) to assess the severity of dementia. The other seven studies did not clearly state the measurements of diagnostic eligibility criteria [40, 46, 51, 53–55, 57].

#### Prognostic criteria

Nine studies included prognostic eligibility criteria [37, 38, 40, 45, 48, 50, 51, 60, 61]. The ‘surprise question’ was used in four studies [38, 51, 60, 61]. The question is, “Would I be surprised if this patient died in the next 12 months?”, which has been used to identify patients at a high risk of death who might benefit from palliative care services [106]. Three [45, 60, 61] used HF-specific standardised prognostic measures. Ng et al. [60] and Wong et al. [61] and used multi-components of the prognostic indicator guidance to identify end-stage heart failure (ESHF) [107]. The indicators are constituted by three steps, which initiate intuitive surprise questions, followed by general and specific clinical indicators. In the three steps, they used only the last step, heart disease-specific clinical indicators. Rogers et al. [45] used the North American Evaluation Study of Congestive Heart Failure and Pulmonary Artery Catheterization Effectiveness (ESCAPE) risk score which uses clinical information to derive a discharge model for six months risk of rehospitalisation and mortality [108]. Bassi et al. [37] used the gender-age-physiology (GAP) index to estimate prognosis and enrol participants with advanced ILD [109]. Helgeson et al. [40] used APACHE and SOFA scoring models to measure severity of critically ill patients admitted to ICU and to predict their mortality [110].

#### Medical history-based criteria

We identified 15 articles that included medical history and treatment criteria. Among 15, eleven trials included criteria related to repeated unplanned hospital admissions due to the deterioration of illness [35, 39, 40, 43–45, 50, 58–61]. The study of dementia by Ahronheim et al. [35] used medical history-based eligibility criteria for hospitalization for acute illness occurred with advanced dementia. However, as many participants died when they were admitted to a hospital, the authors assert these criteria limited study recruitment and attainment of sample size. Treatment-based criteria included previous/current administrative data, such as intravenous therapy support (e.g., diuretics), intubation or non-invasive ventilation, required diuretic dosing, required long-term oxygen therapy, post-cardiac arrest, and results of heart function [36, 48, 51]. Evans et al. [53] considered increasing health service use as a concern caused by severely affected non-malignant chronic conditions.

#### Other criteria

We found four other criteria which can be considered as psychosocial eligibility and needs perceived by healthcare professionals. Higginson et al. [57] asked patients their willingness to engage with a breathlessness support service. Farquhar et al. [54, 55] assessed whether patients might benefit from a self-management programme. Although Helgeson et al. [40] considered the medical ICU perceived need, they did not clearly state the measurements of the criteria.

## Discussion

### Main findings of the study

We systematically reviewed and narratively synthesised the eligibility criteria of 27 RCTs in palliative care. The findings of our review inform development of needs-based triggers for timely referral to palliative care for older adults severely affected by noncancer conditions. The results showed the list of potential needs-based triggers which were composed of three criteria; symptoms, functional status, and QOL. Eligibility criteria that were ‘successful’ tended to utilize at least one domain of needs-based criteria. Six studies used successful eligibility criteria according to the recruitment, attrition rate, and effect on primary outcomes in each study [50, 51, 53, 54, 57, 61].

### What this study adds

Decisions about informing palliative care should be based on individual needs related to symptoms, functional status, and QOL. Few studies used standardized measurements with specific cut-offs for symptoms, functional decline, or QOL assessment.

### Symptoms

Comprehensive assessment aligned to philosophy of palliative care is essential to identify patients likely to benefit from receiving palliative care. In this review, trial eligibility criteria are limited to mainly physical symptoms with little consideration of psychological symptoms e.g. anxiety, depression. Outcome measures of specific physical symptoms such as pain are well developed, but psychosocial symptoms are liable to be considered less serious than physical symptoms. Furthermore, only one study used standardised measures to assess symptom control: MRC dyspnoea scale [57]. Four studies [54, 55, 60, 61] measured physical symptoms such as breathlessness in spite of optimisation of underlying illness, though the measurements are not standardised tools. It implies that using a validated measure for palliative care referral is uncommon.

The best practice of symptom assessment is patient self-report outcomes for example using patient-reported outcome measures (PROMs/PROs) rather than clinician assessment due to the subjective nature of symptoms [111]. However, considering the illness trajectory and deterioration in physical/cognitive abilities in palliative care populations and the potential burden of completing PROMs, reporting by proxies such as relatives or healthcare professionals is important, especially for older adults [112–114]. For older adults with noncancer conditions, loss of mental capacity is common with advancing age associated with for example severe dementia and nearness to end of life. Therefore, measures used in palliative care need to be validated for the population and clinical practice, and for both self and proxy reporting [115]. Some outcome measures include a proxy version, for example the Integrated Palliative Care Outcome Scale (IPOS) [116]. This allows for the adjusting of proxy ratings if the patient is not able to complete the measure as their disease progresses.

### Functional status and quality of life

Functional status and QOL are important needs-based triggers for older adults with noncancer conditions. Functional status is defined as the level of ability to do “activities performed by an individual to realize the needs of daily living in many aspects of life including physical, psychological, spiritual, intellectual, and roles [117]. Three of the included trials indicate that using standardised measures to assess functional status of patients is important to identify individuals likely to benefit from palliative care [35, 36, 51]. Most older adults severely affected by noncancer conditions experience progressive functional disability and subsequent health decline during the course of their disease. Moreover, some studies reported that functional status is significantly associated with health-related QOL (HRQOL) in people with noncancer conditions [118, 119]. Although disease-specific functional assessment measures can be available in some noncancer diseases, the Australia-modified Karnofsky Performance Status (AKPS) is a modified version for palliative care that is widely used and appropriate for multiple care settings in palliative care populations [120]. Collecting and evaluating data on functional status during routine care could inform the need for palliative care for timely referral.

The primary goal of palliative care for older people is to improve QOL with provision based on their needs [13, 29]. Quality of life can be defined as a complex, multifaceted construct that requires multiple approaches from different theoretical angles [121]. Although physical and psychological symptoms and functional impairment can be related to decline in QOL, QOL can be a trigger for referral as it can reflect an unmet need. In this review, eligibility criteria related to QOL were uncommon. As QOL that has a broad multidimensional concept can be difficult to be used as a single referral criterion, it could be operationalized as referral criteria in conjunction with other needs-based criteria. There are few relevant assessment tools addressing functional status and HRQOL for populations with multiple chronic conditions [122].

### Willingness to engage with intervention

Psychosocial eligibility criteria were uncommon, mostly limited to views on willingness to engage with the intervention [54, 55, 57]. One of the major differences between palliative care and other fields of healthcare is the holistic approach it takes, including psychosocial and spiritual dimensions in addition to physical suffering. Willingness to receive palliative care may reflect the patient's preference and could form a needs-based trigger for a referral on preference for palliative care. However, patients who have preferences for palliative care may differ in characteristics compared to those with reluctance to refer to palliative care. For example, low level of health literacy of illness may preclude understanding on benefit of receiving palliative care service and impede individuals’ access to palliative care services. The Health Literacy Skills conceptual framework introduced by Squires et al. [123] illustrates mediators between health literacy and health outcomes. According to the framework, lack of knowledge about available palliative care services means patients do not request or access these services [124, 125]. Educating individuals about the role and function of palliative care, and confirming the willingness to engage with the intervention, may be one of the simplest ways to assess needs-based triggers for a referral on preference for palliative care.

### Limited availability of validated assessment tools

A barrier to using individual needs-based triggers for referral criteria is the limited availability of validated and brief standardised assessment tools encompassing symptom severity, functional status, and QOL for older people with noncancer conditions. Whilst generic measures are able to be used on a large range of health and in various health conditions and populations, specific measures specifically developed to measure outcomes in palliative care are more responsive to needs-based triggers than generic outcome measures. As palliative care focuses on providing holistic care, the outcome measure used to assess palliative care needs for people with noncancer diseases should be comprehensive and encompass multiple health domains [126, 127]. Validated comprehensive measures for palliative care are available and used in clinical care, for example Edmonton Symptom Assessment Scale (ESAS) [128], and the IPOS [116] with condition specific measure for dementia and multi-morbidity (IPOS-Dem) [129]. They encompass holistic domains including, physical, psychological, social and spiritual dimensions.

### Implications for further research and practice

Our review produced the initial step toward developing standardized referral criteria for clinical practice for older adults severely affected by noncancer conditions. Although the results inform a needs-based set of triggers for timely referral to palliative care, further research is needed to examine the feasibility, outcome and processes to operationalise the needs-based triggers as referral criteria in clinical settings.

Future research is needed to develop an international consensus on referral criteria for older adults with noncancer conditions and investigate if the developed referral criteria can be used in clinical settings to identify patients likely to benefit from receiving palliative care. The provision of palliative care should be based on needs assessment [32]. Our findings indicate that needs-based criteria are more likely to suit older people with noncancer conditions. Suitable needs-based referral criteria should meet the varied needs of people with different illness trajectories and different complexities of need for palliative care [32]. We recommend using measurements that encompass symptoms, QOL and functional status. Simple comprehensive measures developed and validated for palliative care population are practical for quick assessment of the palliative care needs, for example IPOS [116] or ESAS [128]. As palliative care needs vary widely, continued assessment of needs-based triggers are advocated. Standardised measures that can aid clinicians to assess palliative needs and concerns should be easy to use and interpret for all healthcare professionals and short to accommodate time constraints in clinical settings [130].

### Strengths and limitations of the study

A key strength of this review is the identification and analysis of trial eligibility criteria for noncancer conditions without restricting by diagnosis. This intended to identify referral criteria applicable across noncancer conditions and multimorbidity. However, most participants in the included studies had heart failure or chronic respiratory disease. This strengthens the applicability of the identified needs-based set of triggers for these population groups, but may limit wider application to all older patients with other noncancer conditions. There is great heterogeneity among older people aged over 65 driven by for example variable diagnosis and multimorbidity, compared to any other age group. The impact this of heterogeneity on the recommendations for palliative care referral should be carefully considered. Though some studies that included mixed diagnosis attempted to reduce the heterogeneity of multimorbidity by identifying disease combinations, future research should consider how to manage heterogeneity, including stratification by age, diagnostic group, and number of co-morbidities. In the study selection process, although we assessed all relevant full-articles by two reviewers independently, the titles and abstracts of studies retrieved in bibliographic searches were assessed by one reviewer. Single screening of the titles and abstracts can influence the number of studies missed. Finally, the included studies were predominantly conducted in high-income countries in Europe and the US. This limits generalisability to non-Western regions and low-middle income countries.

## Conclusion

The findings of this systematic review and narrative synthesis inform development of needs-based triggers for timely referral to palliative care for older people severely affected by noncancer conditions. For older people severely affected by noncancer conditions, decisions about providing palliative care should be based on the present needs related to symptoms, functional status, and quality of life. Further research is needed to examine the feasibility, outcome and processes to operationalise the needs-based triggers as referral criteria in clinical settings and develop international consensus on referral criteria for older adults with noncancer conditions.

## Supplementary Information

**Additional file 1.** 

**Additional file 2.** 

**Additional file 3.** 

**Additional file 4.**

## Data Availability

The PRISMA 2020 checklist, the full search strategy, and the risk of bias plots have been presented in Additional files. Any further data analysed during this study are available from the corresponding author on reasonable request.

## Abbreviations

AKPS: The Australia-modified Karnofsky Performance Status
APACHE: The Acute Physiology and Chronic Health Evaluation
CHF: Chronic Heart Failure
COAD: Chronic Obstructive Airways Disease
COPD: Chronic Obstructive Pulmonary Disease
CRD: Centre for Reviews and Dissemination
CRQ: The Chronic Respiratory Disease Questionnaire
EAPC: European Association for Palliative Care
EDSS: Emergency Department
ESAS: Edmonton Symptom Assessment System
ESCAPE risk score: The Evaluation Study of Congestive Heart Failure and Pulmonary Artery Catheterization Effectiveness risk score
ESHF: End-Stage Heart Failure
FAST: Functional Assessment Staging Tool
GAP: The Gender-Age-Physiology index
GDS: The Global Deterioration Scale
GOLD: The Global Initiative for Chronic Obstructive Lung Disease
HF: Heart Failure
HRQOL: Health-related quality of life
ICU: Intensive Care Unit
ILD: Interstitial Lung Disease
IPF: Idiopathic pulmonary fibrosis
IPOS: The Integrated Palliative care Outcome Scale
KCCQ: Kansas City Cardiomyopathy Questionnaire Short Version
MRC: Medical Research Council
MRQr: Maugeri Respiratory Questionnaire
NYHA: New York Heart Association
PD: Parkinson’s Disease
PC-NAT: The Palliative Care Needs Assessment Tool
PDQ: The Parkinson's Disease Questionnaire
PRISMA: Preferred Reporting Items for Systematic Reviews and Meta-analyses
PROMs: Patient Reported Outcome Measures
PROSPERO: The International Prospective Register of Systematic Reviews
QoL-AD: The Quality of Life in Alzheimer’s Disease
RCT: Randomised Controlled Trial
SGRQ: The St. George’s Respiratory Questionnaire
SOFA: The Sequential Organ Failure Assessment
VAS: Visual Analogue Scale
