# Supplementary material for: Needs-based triggers for timely referral to palliative care for older adults severely affected by noncancer conditions: a systematic review and narrative synthesis
Source: BMC Palliat Care. 2023 Mar 9;22:20. doi: 10.1186/s12904-023-01131-6 (PMC9996955; doi:10.1186/s12904-023-01131-6)
Supplement: Supplementary file 2 — Additional file 2. [file 12904_2023_1131_MOESM2_ESM.docx]

**Additional file 2 – Search strategy**

**MEDLINE search strategy via OVID 1946 to September 14, 2018**

1. randomized controlled trial.pt.

2. controlled clinical trial.pt.

3. randomized.ab.

4. clinical trials as topic.sh.

5. randomly.ab.

6. trial.ti.

7. 1 or 2 or 3 or 4 or 5 or 6

8. exp terminally ill/

9. exp frailty/

10. (EOL or End-of-life or dying or LYOL or (last adj4 life)).mp.

11. (life adj (limit* or threat*)).mp.

12. ((advance* or terminal* or progressive) adj (disease* or ill*)).mp.

13. (multi*morbidity or co*morbidity).mp.

14. ((final or last or end) adj stage*).mp.

15. (frail old* and (people or adult* or person*)).mp.

16. frail*.mp.

17. frail elder*.mp.

18. frailty syndrome*.mp.

19. 8 or 9 or 10 or 11 or 12 or 13 or 14 or 15 or 16 or 17 or 18

20. exp Terminal care/

21. exp Palliative Care/

22. (palliative or palliation).mp.

23. (palliative adj (treatment or medicine or care*)).mp.

24. (special* adj2 palliat*).mp.

25. ((terminal or supportive) adj care*).mp.

26. 20 or 21 or 22 or 23 or 24 or 25

27. 7 and 19 and 26

**Embase search strategy via OVID 1974 to 2018 Week 38**

1. randomized controlled trial.de.

2. controlled clinical trial.de.

3. randomized.ab.

4. exp "clinical trial (topic)"/

5. randomly.ab.

6. trial.ti.

7. 1 or 2 or 3 or 4 or 5 or 6

8. exp terminal disease/

9. exp terminally ill patient/

10. (EOL or End-of-life or dying or LYOL or (last adj4 life)).mp.

11. (life adj (limit* or threat*)).mp.

12. ((advance* or terminal*) adj (disease* or ill*)).mp.

13. (multi*morbidity or co*morbidity).mp.

14. (frail old* and (people or adult* or person*)).mp.

15. frail*.mp.

16. frail elder*.mp.

17. frailty syndrome*.mp.

18. 8 or 9 or 10 or 11 or 12 or 13 or 14 or 15 or 16 or 17

19. exp Terminal care/

20. exp palliative therapy/

21. (palliative or palliation).mp.

22. (palliative adj (treatment or medicine or care*)).mp.

23. (special* adj2 palliat*).mp.

24. ((terminal or supportive) adj care*).mp.

25. 19 or 20 or 21 or 22 or 23 or 24

26. 7 and 18 and 25

**PsycINFO search strategy via OVID 1806 to September Week 2 2018**

1. randomized controlled trial.mp.

2. controlled clinical trial.mp.

3. randomized.ab.

4. exp Clinical Trials/

5. randomly.ab.

6. trial.ti.

7. 1 or 2 or 3 or 4 or 5 or 6

8. exp Terminally Ill Patients/

9. (EOL or End-of-life or dying or LYOL or (last adj4 life)).mp.

10. (life adj (limit* or threat*)).mp.

11. ((advance* or terminal*) adj (disease* or ill*)).mp.

12. (multi*morbidity or co*morbidity).mp.

13. (frail old* and (people or adult* or person*)).mp.

14. frail*.mp.

15. frail elder*.mp.

16. frailty syndrome*.mp.

17. 8 or 9 or 10 or 11 or 12 or 13 or 14 or 15 or 16

18. exp PALLIATIVE CARE/

19. (palliative or palliation).mp.

20. (palliative adj (treatment or medicine or care*)).mp.

21. (special* adj2 palliat*).mp.

22. ((terminal or supportive) adj care*).mp.

23. 18 or 19 or 20 or 21 or 22

24. 7 and 17 and 23

**CINAHL search strategy via EBSCOhost**

S24S6 AND S16 AND S23

S23S17 OR S18 OR S19 OR S20 OR S21 OR S22

S22TX (terminal or supportive) N1 care*

S21TX special* N2 palliat*

S20TX palliative N1 (treatment or medicine or care*)

S19TX palliative or palliation

S18(MH "Terminal Care")

S17(MH "Palliative Care")

S16S7 OR S8 OR S9 OR S10 OR S11 OR S12 OR S13 OR S14 OR S15

S15TX frailty syndrome*

S14TX frail elder*

S13TX frail*

S12TX frail old* and (people or adult* or person*)

S11TX (multimorbidity or comorbidity)

S10TX (advance* or terminal*) N1 (disease* or ill*)

S9TX life N1 (limit* or threat*)

S8TX EOL or End-of-life or dying or LYOL or (last N4 life)

S7(MH "Terminally Ill Patients")

S6S1 OR S2 OR S3 OR S4 OR S5

S5TI trial

S4AB randomly

S3AB randomized

S2(MH "Clinical Trials")

S1(MH "Randomized Controlled Trials")

**CENTRAL search strategy**

#1randomized controlled trial.pt.

#2controlled clinical trial.pt.

#3randomized.ab.

#4clinical trials as topic.sh.

#5randomly.ab.

#6trial.ti.

#7#1 or #2 or #3 or #4 or #5 or #6

#8MeSH descriptor: [Terminally Ill] explode all trees

#9MeSH descriptor: [Frailty] explode all trees

#10EOL or End-of-life or dying or LYOL or (last adj4 life)

#11life adj (limit* or threat*)

#12(advance* or terminal* or progressive) adj (disease* or ill*)

#13(multi*morbidity or co*morbidity).ti,ab.

#14(final or last or end) adj stage*

#15frail old* and (people or adult* or person*)

#16frail*

#17frail elder*

#18frailty syndrome*

#19#8 or #9 or #10 or #11 or #12 or #13 or #14 or #15 or #16 or #17 or #18

#20MeSH descriptor: [Palliative Care] explode all trees

#21MeSH descriptor: [Terminal Care] explode all trees

#22palliative or palliation

#23palliative adj (treatment or medicine or care*)

#24special* adj2 palliat*

#25(terminal or supportive) adj care*

#26#20 or #21 or #22 or #23 or #24 or #25

#27#7 and #19 and #26

**Clinical trials. Gov**

**Applied Filters: Condition or disease: “Palliative care”, Age group: Adult (18-64) plus Older Adult (65+), Study type: Interventional Studies (Clinical Trials)**

**(Search result), (Entire Database)**

palliative care

Symptom Management

Palliation

Palliative Treatment

Palliative Therapy

Comfort Care
