# Supplementary figures and images for: Needs-based triggers for timely referral to palliative care for older adults severely affected by noncancer conditions: a systematic review and narrative synthesis
Source: BMC Palliat Care. 2023 Mar 9;22:20. doi: 10.1186/s12904-023-01131-6 (PMC9996955; doi:10.1186/s12904-023-01131-6)

**Additional file 3 – Risk of Bias: Traffic-light plot**


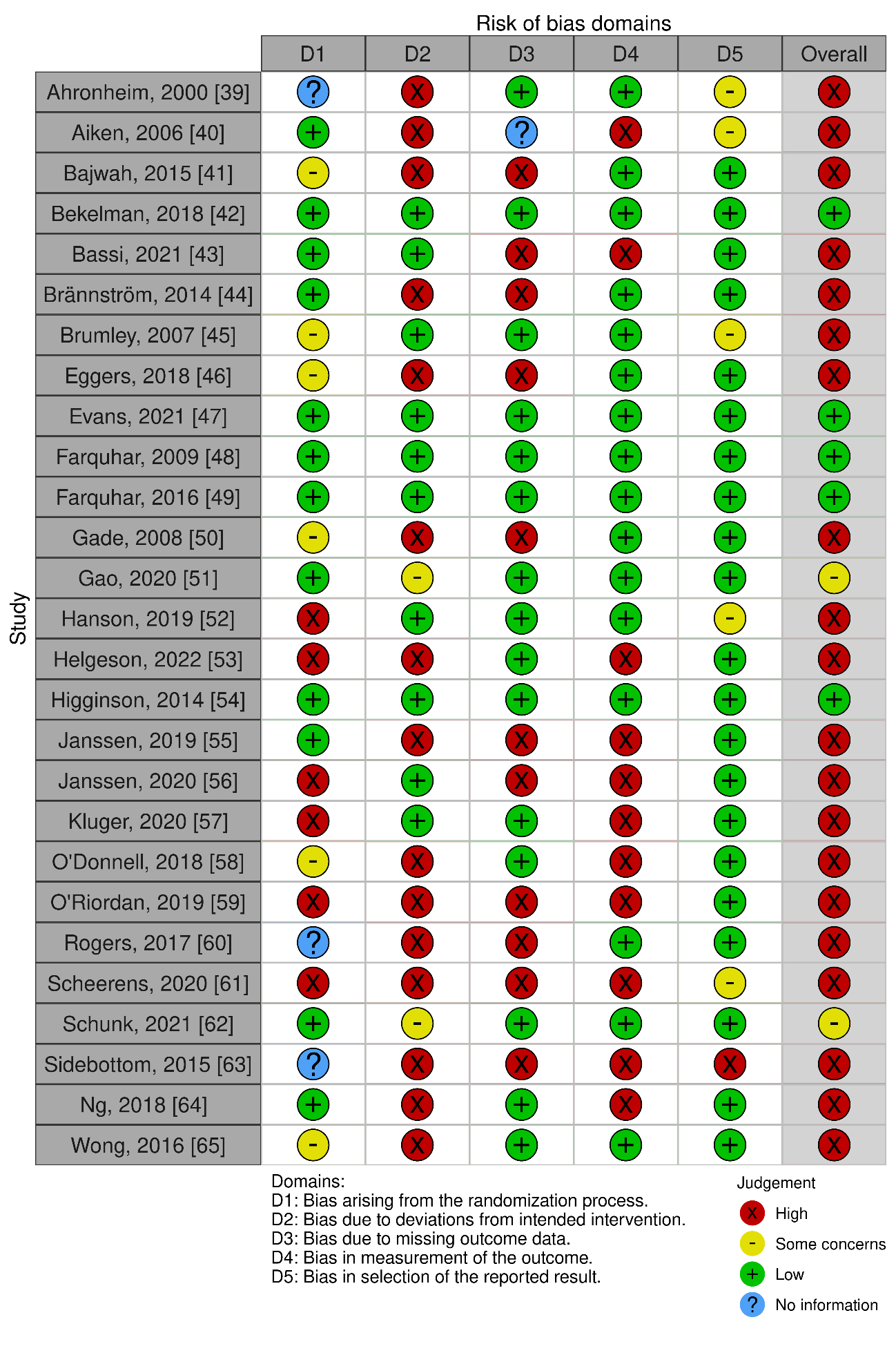

Supplement: Supplementary file 3 — Additional file 3. [file 12904_2023_1131_MOESM3_ESM.docx]

**Additional file 4 – Risk of Bias: Summary plot**

**
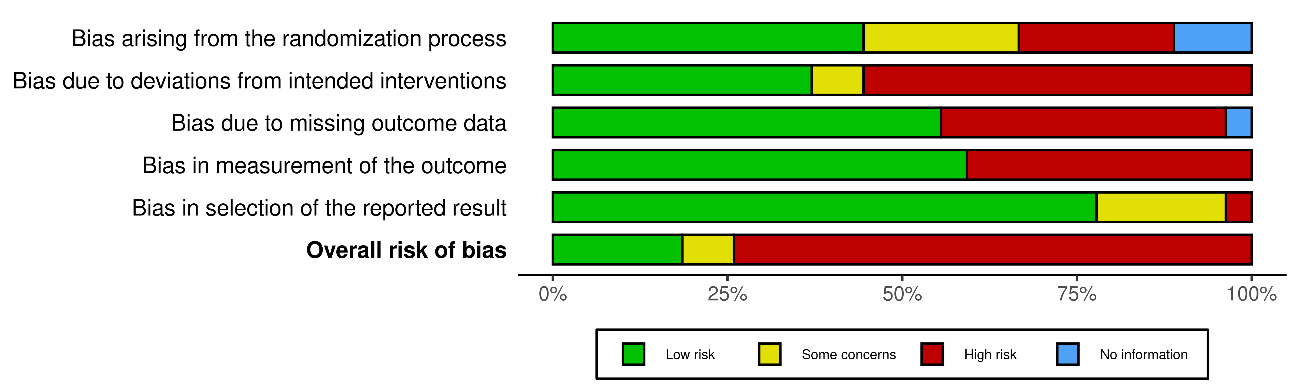
**

Supplement: Supplementary file 4 — Additional file 4. [file 12904_2023_1131_MOESM4_ESM.docx]
